# The Role of microRNAs in the Gonocyte Theory as Target of Malignancy: Looking for Potential Diagnostic Biomarkers

**DOI:** 10.3390/ijms231810526

**Published:** 2022-09-10

**Authors:** Fabiola García-Andrade, Rosa María Vigueras-Villaseñor, Margarita Dolores Chávez-Saldaña, Julio César Rojas-Castañeda, Iván Uriel Bahena-Ocampo, Elena Aréchaga-Ocampo, José Díaz-Chávez, Daniel Adrian Landero-Huerta

**Affiliations:** 1 Laboratorio de Biología de la Reproducción, Instituto Nacional de Pediatría, Ciudad de México 04530, Mexico; 2 Posgrado en Biología Experimental, Universidad Autónoma Metropolitana Unidad Iztapalapa, Ciudad de México 09310, Mexico; 3 Departamento de Ciencias de la Salud, Universidad Autónoma Metropolitana Unidad Iztapalapa, Ciudad de México 09310, Mexico; 4 Departamento de Ciencias Naturales, Universidad Autónoma Metropolitana Unidad Cuajimalpa, Ciudad de México 05348, Mexico; 5 Instituto Nacional de Cancerología, Ciudad de México 14080, Mexico

**Keywords:** microRNAs, gonocytes, germ cell neoplasia in situ (*GCNIS*), cryptorchidism, testicular germ cell tumor

## Abstract

Some pediatric patients with cryptorchidism preserve cells with gonocyte characteristics beyond their differentiation period, which could support the theory of the gonocyte as a target for malignancy in the development of testicular neoplasia. One of the key molecules in gonocyte malignancy is represented by microRNAs (miRNAs). The goal of this review is to give an overview of miRNAs, a class of small non-coding RNAs that participate in the regulation of gene expression. We also aim to review the crucial role of several miRNAs that have been further described in the regulation of gonocyte differentiation to spermatogonia, which, when transformed, could give rise to germ cell neoplasia in situ, a precursor lesion to testicular germ cell tumors. Finally, the potential use of miRNAs as diagnostic and prognostic biomarkers in testicular neoplasia is addressed, due to their specificity and sensitivity compared to conventional markers, as well as their applications in therapeutics.

## 1. Introduction

The genome contains all the hereditary information of an organism that specifies the genetic instructions for its development and functioning. However, only 1.5% of the information transmitted transversely has a coding function, while the rest includes spacer and regulatory regions, as well as many other sequences with unknown function [1]. In recent years, with the introduction of omic sciences, the study of gene expression using microarray expression assays, and whole-transcriptome sequencing, it has been shown that at least 90% of the genome is actively transcribed and that the human transcriptome is more complex than the set of genes that encode proteins [2]. MicroRNAs (miRNAs) are small non-coding RNAs that participate in the regulation of the expression of a large number of genes in multiple biological processes at the post-transcriptional level [3]. miRNAs can have tissue- and organ-specific expression patterns and can even be used as a powerful tool for histological classification in tumor samples of unknown origin [4]. While single-cell expression patterns of messenger RNAs can distinguish different cell populations and their states of differentiation in the testis, miRNAs may be more specific compared to transcript analyses [5].

In particular, in gonocytes, miRNAs regulate the maintenance of pluripotency, apoptosis, cell metabolism, and their differentiation into spermatogonia, essential processes that ensure fertility and prevent the development of testicular neoplasia in adulthood [6].

It has been proposed that gonocytes are responsible for the development of testicular neoplasia, because these cells and atypical cells of germ cell neoplasia in situ (*GCNIS*) share morphological characteristics and protein expression patterns, such as KIT, POU5F1, AP-2γ, and SALL4 [7,8]. *GCNIS* is considered to be a precursor lesion for the development of testicular germ cell tumors (TGCT) [9], because it has been reported that patients with *GCNIS* develop TGCT within 5 years [10]. Furthermore, common epigenetic markers have also been described between the cells that constitute *GCNIS* and gonocytes, finding elevated expression of H3K4me, H2A.Z, H3K9ac, and H4/H2A R3me2 and decreased expression of 5mC, H3K9me2, and H3K27me3, which are related to more open chromatin and low levels of DNA methylation [11].

In addition, in recent years, the persistent presence of gonocytes and the abnormal expression of their typical markers have been described in some patients with cryptorchidism (CO) [12], where they are associated with high proliferative activity and the expression of gene markers related to neoplastic transformation [11]. This suggests that these cells are unable to differentiate into spermatogonial stem cells (SSC), and, in turn, a proportion of these gonocytes do not follow the normal process of apoptosis in the testis [7]. This explains why patients with CO have a higher risk of developing TGCT in adulthood compared to the general population [13].

Currently, little information exists on the differential expression of various miRNAs in both *GCNIS* and CO [14,15]. Conversely, in the case of gonocytes and TGCT, the information is robust [16,17]. Due to the high specificity and sensitivity of the hsa-miR-371-373 and hsa-miR-302/367 clusters as biomarkers for the diagnosis and prognosis of TGCT, in the future, these miRNAs could displace classical markers such as lactate dehydrogenase (LDH), human alpha-fetoprotein (AFP), and human chorionic gonadotropin (HCG) [18,19]. 

Based on this information, this work recapitulates various studies of miRNA expression in cancer, and considers how regulation by these miRNAs could influence gonocyte persistence, possibly resulting in a pathological outcome, such as *GCNIS* and CO, which finally culminates in the development of TGCT in adulthood.

## 2. General Characteristics of miRNAs

miRNAs are a class of single-stranded non-coding RNAs (ncRNAs) with an average length of between 19 and 25 nucleotides. They regulate the abundance of a number of mRNAs at the post-transcriptional level. miRNAs generally bind to the 3′-UTR region of their target mRNAs, inducing their degradation or inhibiting translation [20,21]. Thus far, approximately 1917 miRNA precursors and 2654 mature miRNA sequences have been reported in humans. It is currently estimated that approximately 60% of genes are probably regulated by miRNAs [22,23]. miRNAs can be found in intergenic or intronic regions, approximately between 52% and 40%, respectively, while the remaining 8% are found in exonic regions [24]. The expression of miRNAs found in intergenic zones is produced by their own regulatory elements, while the expression of those found in intragenic zones (intronic or exonic) is dependent on the expression machinery of the host gene [25]. Additionally, miRNAs can be generated from a single precursor or from a common precursor forming clusters, expressed jointly and then being split into individual miRNAs, regulating genes with related functions [26]. 

Each miRNA can regulate hundreds of mRNAs, while one mRNA can be regulated by several miRNAs, having several binding sites in the UTR region. Notably, the miRNA–mRNA interactions that determine the post-transcriptional regulation of a gene are conserved between homologous genes and homologous miRNAs, establishing regulatory networks conserved in evolution [27].

**Figure 1:**
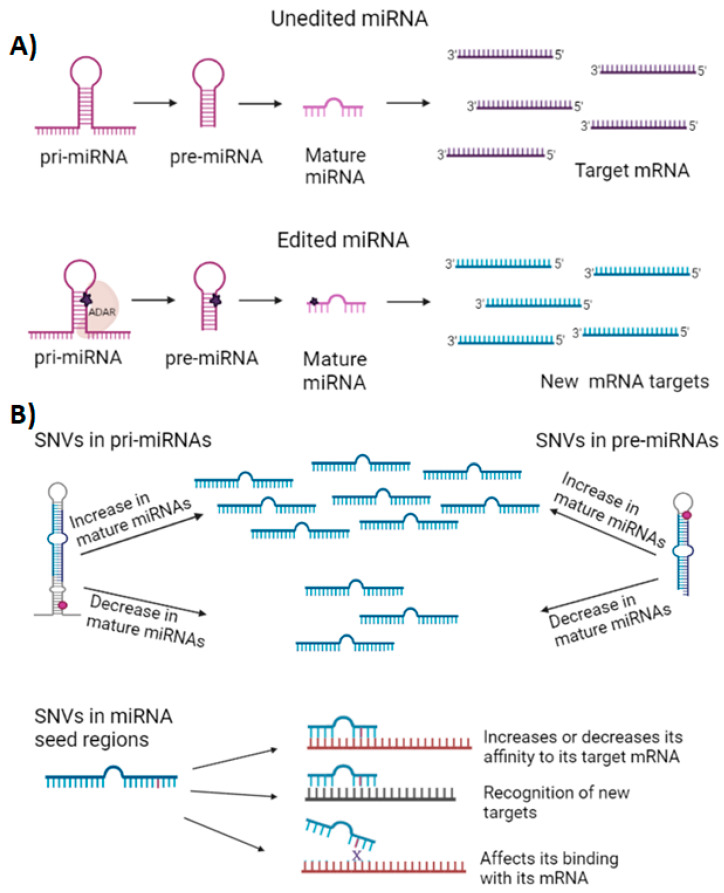
Mechanisms of miRNA regulation by nucleotide modification. (**A**) Pri and pre-miRNA precursors that undergo ADAR sequence modifications from adenosine to inosine, affecting their maturation, binding to the RISC complex, or redirection to other new target mRNAs. (**B**) Representation of SNVs in pri-miRNAs or pre-miRNAs that can alter miRNA processing, secondary structure, and splicing, causing an increase or decrease in mature miRNAs, as well as SNVs in the miRNA seed region. These modifications can increase or decrease affinity to their target genes, recognize new target genes, or inhibit binding to their target genes. Created with Biorender.com.

miRNAs can also be regulated by nucleotide modifications mediated by the RNA-acting adenosine deaminase (ADAR) family of proteins, which edit adenosines to inosines (A to I) in miRNA precursors, disrupting miRNA–target mRNA binding and redirecting them to a different target, as shown in Figure 1A. In addition, the cytidine deaminase/apolipoprotein B-induced activation protein family of cytidine deaminase mRNA editing enzymes (AID/APOBEC) can convert cytidine to uridine (C to U), thus generating cytidine variants. These miRNAs, called “isomiRs”, can regulate the same or different target mRNAs [28,29]. Another mechanism that can regulate miRNA expression is the presence of single-nucleotide variants (SNVs), which affects the secondary structure of the miRNA, as well as the maturation process and their functions [30]. SNVs within the miRNA seed region sequence can lead to the gain or loss of interactions with their target mRNA, while SNVs in the 3′-UTR of mRNA can likewise affect miRNA–mRNA binding, as shown in Figure 1B [31]. 

**Figure 2:**
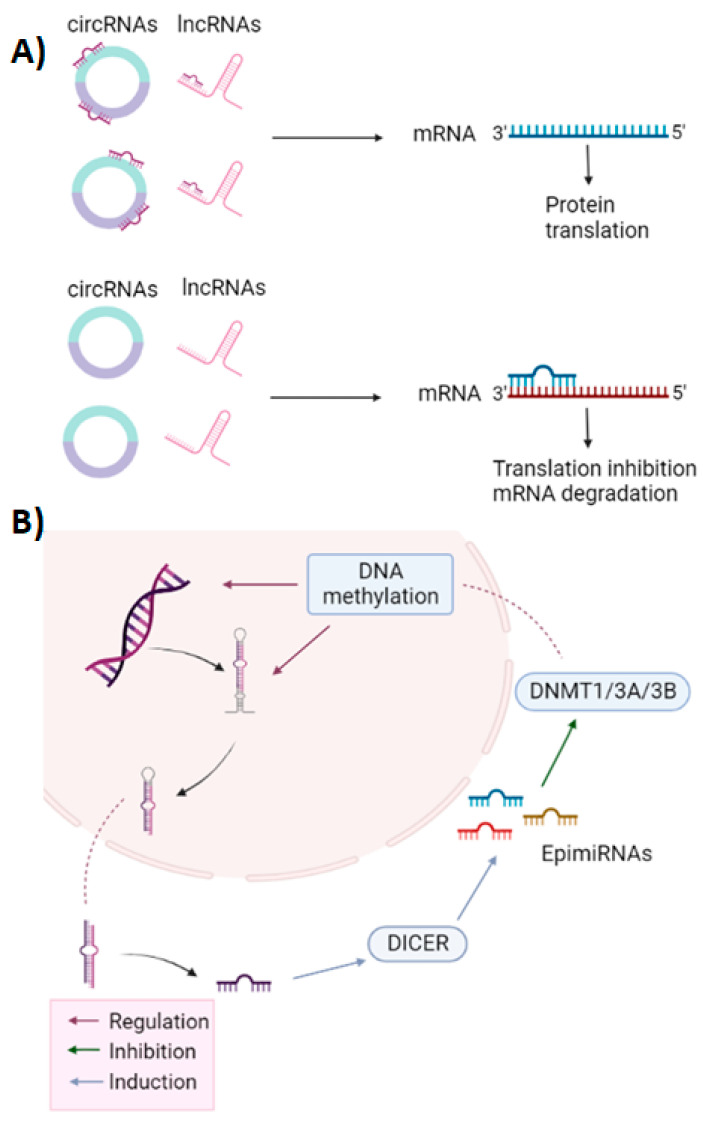
Other mechanisms of miRNA regulation. (**A**) epi-miRNAs are involved in the regulation of epigenetic factors associated with DNA methylation, such as DNMT1, DNMT3A, DNMT3B, which participate in the regulation of expression of some miRNAs that, through DICER, regulate epi-miRNAs, giving rise to a regulatory loop that is frequently altered in cancer. (**B**) Competing endogenous RNAs (ceRNAs), such as some long non-coding RNAs (lncRNAs) and circular RNAs (circRNAs), affect the interactions of miRNAs with their target mRNA, competitively sequestering miRNAs, reducing the repression of target mRNAs. Created with Biorender.com.

Another important mechanism for regulating miRNA expression is epigenetic control, such as DNA methylation or acetylation, as well as histone and chromatin modifications. These are the main reasons for alterations in miRNA expression in cancer. Some miRNAs can participate as epigenetic regulators called “epi-miRNAs” that regulate the expression of DNA methyltransferases (DNMT), histone deacetylases (HDAC), and histone methyltransferases (HMT), affecting gene expression, as shown in Figure 2A [32,33]. The interaction of miRNAs with their target mRNAs can also be altered by the expression of other RNAs, such as circular RNAs (circRNA) and lncRNA, which participate as endogenous competitors (ceRNA) of some miRNA targets, as shown in Figure 2B [34,35].

miRNAs are normally involved in multiple biological functions and cellular processes, as well as in the development of various pathologies, such as cancer. An example of this is cell to cell communication through packing in exosomes or binding proteins that are transported to the extracellular environment and then are taken by other cells [3,35]. Some miRNAs can be transported to the nucleus and bind to double-stranded DNA to modulate the repression of target genes [36], while others can regulate the transcription and functions of their host gene [25].

In addition, miRNAs can present cell-type and/or tissue-dependent expression, and alteration of this specificity is related to different tumors [37]. Alterations of the miRNA expression in tumors allow also alterations in the regulation of different mRNAs. According to the targets of tumor suppressor genes or oncogenes, miRNAs could be classified as oncomiRs or tumor suppressor miRNAs. They can regulate initiation and progression, as well as the development of metastasis, which implies their functional role in migration, invasion, evasion of the immune response, and uncontrolled cell proliferation [38,39]. miRNAs also participate in other fundamental processes, such as embryonic differentiation, in the development of the male germ line, and in spermatogenesis, strictly regulated processes, both at the transcriptional and post-transcriptional levels [40].

## 3. From the Gonocyte to TGCT

### 3.1. miRNAs Expressed in Primordial Germinal Cells/Gonocytes

The testicle is a fundamental organ, where male gametes are produced. Gonadal somatic cells are responsible for both normal gonadal development and sex determination, as well as guiding the development of germ cells. These processes involve interactions of several genes, signaling pathways, and different types of cells, such as primordial germ cells (PGC), which are formed in the proximal epiblast, mediated by bone morphogenetic protein 4 (BMP4) signaling, which then promotes the differentiation and migration of PGCs to the genital ridges, where they proliferate. Once established in the seminiferous cords, they are called gonocytes, which are round cells with a prominent nucleus and condensed nucleolus located in the center of the seminiferous cords. At birth and until approximately 6 months of life in humans, gonocytes migrate to the basement membrane, resuming their proliferation and differentiation towards spermatogonial stem cells (SSCs) [41,42]. It has been reported that the activation of the hypothalamic–pituitary–testis axis is important for the differentiation of gonocytes in the first few months of life, during the process called “mini puberty”; thus, androgen and gonadotropin deficiencies alter this process [43]. 

In this sense, some miRNAs differentially expressed in gonocytes have been identified in both mice and humans. Some of these are the mmu-miR-290-295 cluster, mmu-miR-136, mmu-miR-743a, and mmu-miR-463-3p. The expression of these miRNAs changes during gonocyte differentiation to spermatogonia, and they participate in signaling pathways related to *Cxcr4*, *Pten*, and *Wnt*/β, associated with TGCT development [16]. The mmu-miR-290-295 cluster is important for the migration and survival of mouse embryonic stem cells, participating in proliferation, and, together with the miR-302/367 cluster, is exclusively expressed in undifferentiated pluripotent stem cells and is involved in embryonic development in both mice and humans [44,45]. The mmu-miR-290-295 cluster regulates the expression of pluripotency genes such as *Pou5f1*, *Sox2*, and *Nanog* [16], and its orthologous cluster, hsa-miR-371-373, is overexpressed in embryonic stem cells. Later, during the differentiation of these cells, there is decreased expression of these miRNAs [46,47]. Other miRNAs that are overexpressed during the development of primordial germ cells are the mmu-miR-17-92 cluster, mmu-let-7, mmu-miR-125a, and mmu-miR-9. In turn, miR-19a and miR-19b are conserved between the mouse and human, and participate in the proliferation of primordial germ cells by regulating the *PTEN* gene. Furthermore, hsa-miR-141-/200c, in both the mouse and human, perform similar functions and are believed to be involved in the development of TGCT in humans [48,49].

In addition, it has been reported that some transcription factors such as Pou5f1/POU5F1 can bind to promoters and then activate the transcription of mmu-miR-290-295 and hsa-miR-302/367 [50,51]. The mmu-miR-290 cluster is overexpressed in mouse embryonic stem cells, while its counterpart, the hsa-371-373 cluster, is overexpressed in human embryonic stem cells, as is the hsa-miR-302/367 cluster. Both clusters participate in the maintenance of pluripotency. They are also found abundantly during the reprogramming and self-renewal of embryonic stem cells, and finally their expression decreases when these cells differentiate [52]. Other miRNAs that participate in the maintenance of stem cell pluripotency are mmu-miR-203, mmu-miR-369, and mmu-miR-200c, the latter being activated by *Pou5f1* [53]. It has also been reported that the reduction of hsa-miR-145 in humans inhibits the expression of pluripotency genes such as *POU5F1*, *SOX2*, and *KLF4*, inducing differentiation and the inhibition of human embryonic stem cells (hESCs) self-renewal [54]. Conversely, miRNAs such as mmu-miR-134, mmu-miR-470, and mmu-miR-21 inhibit genes related to pluripotency, such as *Nanog*, *Sox2*, and *Pou5f1*, participating in cell differentiation [55]. In the case of mmu-Let-7, mmu-miR-23b, and mmu-miR-21, these participate in the differentiation of primordial germ cells by regulating the *Lin28* and *Blimp1* genes [56].

### 3.2. miRNAs Expressed in Cryptorchidism

CO, or undescended testicle, is a common anomaly in newborn males and can affect one (unilateral) or both (bilateral) testicles [57]. Based on the anatomical location of the testis, CO is classified as (1) abdominal, (2) inguinal, and (3) ectopic [58]. This malformation has a variable prevalence between one population and another, reporting a frequency of 9.0% in Denmark and 2.4% in Finland [59]. Despite extensive knowledge of testicular physiology, the etiology remains largely unknown. However, it is considered to be a multifactorial condition [60], common and of great importance in pediatric age, due to its frequency and possible repercussions in adulthood [61]. This malformation is clinically associated with the development of TGCT [62], since it has been shown that patients with CO have a higher relative risk (RR) of 4.8 (95% confidence interval: 4.0–5.7) for the development of TGCT [63] compared to the general population.

Some reports on the testicular tissue of young patients with CO showed the depletion of Ad spermatogonia in the germinal epithelium, which led the authors of this work to interpret these cells as stem cells of all future spermatozoa [64], in line with the theory that proposes the gonocyte as the precursor of the *GCNIS*, attributed to the failure of differentiation in primordial germ cells [65].

Moreover, it is suggested that failure of mini puberty has an effect on gonocyte maturation; however, this is still under debate. There are studies that show that the maturation of gonocytes is independent of androgens; in addition to this, it was reported in the testicular tissue of patients with undervirilization syndromes that gonocytes appeared to transform earlier and in greater numbers than normally; therefore, a lack of androgens may stimulate non-androgenic regulators to trigger gonocyte transformation, highlighting the need to identify non-androgenic regulatory pathways [66,67,68]. There are no reports of the hormonal effect on the expression of miRNAs in gonocytes from CO patients. However, the effect of GnRh on the expression of some LncRNAs and its possible relationship with the expression of certain genes in the Ad spermatogonia of CO patients has been described [69]. In this report, the authors assume a relationship between some LncRNAs such as *HOTAIR* and *EGFR*-AS1 with the expression of certain genes, but the study lacks a strict correlation due to not validating the results in a greater number of CO patients, and ignoring the functional validation to assert that these LncRANs regulate the target genes that they propose in Ad spermatogonia from CO patients. LncRNAs have been reported to silence the expression of certain miRNAs by sponging them in different tumors [70,71,72], which is interesting since some LncRNAs could probably exert direct action on the silencing of the cluster hsa-miR-371-373 or its homologue mmu-miR-290-295, which are widely characterized in the gonocyte, *GCNIS*, and TGCT.

On the other hand, it is believed that these persistent gonocytes could be the origin of TGCT in patients with CO [73,74,75]. In addition, the overexpression of proteins such as KIT, POU5F1, AP-2γ, and SALL4 has been identified in undescended testes in patients at ages at which they should not be expressed [7]. Thus, it is believed that this neoplasm has a fetal origin [76]. However, the specific mechanisms of the transformation of the gonocyte to *GCNIS* are unknown. 

Huang et al. [77] assessed the expression of miR-34c in the testicular tissue of patients with CO in mice, finding subexpression of miR-34c and the consequent overexpression of its target gene *Nanos2*, which promotes the development of male PGC and causes alteration of spermatogonia homeostasis, affecting spermatogenesis in CO. Therefore, it has been suggested that miR-34c could give information about infertility caused by CO and serve as a treatment target for infertility [77]. hsa-miR-210 is related to infertility and germ cell maturation arrest, participating in spermatogenesis and in the development of CO by regulating the *NR1D2* gene that is overexpressed in patients with CO [78]. Therefore, this miRNA could serve as a biomarker for CO in clinical tests. hsa-miR-7-5p and hsa-miR-519d-3p are related to the *AKT3* gene and are believed to also participate in the development of CO [79]. The hsa-miR-22-5p is overexpressed in patients with CO and regulates *EZH2*, participating in the renewal of SSCs. Therefore, this miRNA could serve as a biomarker of infertility in CO [80].

Interestingly, Tang et al. [14], conducted a study using next-generation sequencing (NGS) and proposed a profile of multiple miRNAs that were differentially expressed in three patients with CO (Table 1). Interestingly, in the supplementary data of this report, hsa-miR-371-373 was downregulated in CO patients, compared to controls, which is to be expected because only some CO patients will be at risk of developing TGCT, as has been proposed in other studies [7]. Notably, to date, no miRNA has been reported related to CO with *GCNIS* or even with TGCT. Therefore, a study of this type could help to determine the molecular relationship between these conditions, with the purpose of finding diagnostic and prognostic biomarkers in this malformation.

### 3.3. miRNAs Expressed in Germ Cell Carcinoma In Situ

*GCNIS* is represented by atypical cells with irregularly shaped nuclei, prominent nucleoli, and clear cytoplasm, aligned in a single row in contact with the basement membrane of the seminiferous tubules. It is considered to be the precursor lesion for TGCT. *GCNIS* cells can be dormant and later transform into cancer cells, arresting the process of spermatogenesis [81,82]. To date, the cause for the malignant transformation of cells to *GCNIS* is unknown. This is a lesion that remains inactive and begins to proliferate in adolescence, possibly due to hormonal stimulation. Therefore, TGCT is considered a late-onset pathology. *GCNIS* shares similar characteristics with PGCs and gonocytes—for example, morphological features and expression of fetal germ cell markers KIT, KITL, POU5F1, PLAP, AP-2γ, and NANOG. The expression of this protein is inhibited when gonocytes differentiate into spermatogonia [9].

Some of the miRNAs expressed in *GCNIS* are the hsa-miR-371-373 and hsa-miR-302/367 clusters, which are also associated with TGCT [15]. Specifically, hsa-miR-371a-3p is the most studied as a biomarker for TGCT and has also been reported in *GCNIS* cells, where it presents higher expression levels compared to healthy cells [15,83].

hsa-miR-141 has also been related to the development of *GCNIS* and its detection, along with hsa-miR-200c, which is expressed in *GCNIS* and not in normal testes [15]. Meanwhile, it has been shown that the hsa-miR-17-92 cluster inhibits the *E2F1* gene, participating in the progression of this precursor lesion [84].

### 3.4. miRNAs Expressed in Testicular Germ Cell Tumors

TGCT, which can develop in one or both testes, are genitourinary neoplasms that comprise a heterogeneous class of solid, malignant tumors that are clinically silent in their early stages. Treatment is generally successful; however, they have a significant morbidity and mortality rate [85]. TGCT occur in young men, between 15 and 44 years of age, and their incidence has been increasing in the last 30 years [86] and is highly variable in different populations, reporting higher incidences in Caucasian populations than in the African and Asian populations [87]. It has been postulated that the genetic background of each population plays an important role in the susceptibility to TGCT [88]. The global incidence of TGCT is 2.8 per 100,000 male live births [89]. TGCT are classified into three types, presenting at different ages in men. Type I includes yolk sac tumors that occur in infants. Type II is the most common type of tumor; it is divided into seminoma TGCT (sTGCT) and non-seminoma TGCT (nsTGCT) that occur in young men between 15 and 44 years of age. Lastly, type III, spermatocytic seminomas, occurs in adults over 50 years of age [90].

The cells that comprise sTGCT have a homogeneous composition and are similar to PGCs and gonocytes, while nsTGCT include embryonal carcinoma, yolk sac tumors, choriocarcinoma, and teratoma. The embryonal carcinoma cells present a similar pattern of expression to undifferentiated stem cells; teratoma cells may contain somatic tissues or extraembryonic derivatives, while yolk sac tumor cells present extraembryonic differentiation. Choriocarcinoma contains cytotrophoblast, trophoblast, and syncytiotrophoblast cells, the latter being more differentiated [9,91]. Another characteristic of seminomas is the hypomethylation profile similar to those of their progenitors, the *GCNIS* cells. In the case of embryonic carcinoma cells, they present hypermethylation similar to that of embryonic stem cells [13].

*GCNIS* is thought to develop from gonocytes that fail to differentiate during the first 6 postnatal months, on average, possibly due to altered signaling or a lack of response to differentiation signals; this event occurs prior to the clinical manifestation of TGCT [92]. Some of the miRNAs that have been found expressed in *GCNIS* and have been reported to be involved in the development of TGCT are the miR-371-373 and miR-302/367 clusters, which are normally expressed in human embryonic stem cells and at high levels in TGCT, independent of histological subtype, anatomical site, or patient age [93]. Additionally, they are considered sensitive and specific biomarkers for TGCT prognosis [94]. However, the function of many other miRNAs related to TGCT has also been reported (Table 2).

**Figure 3:**
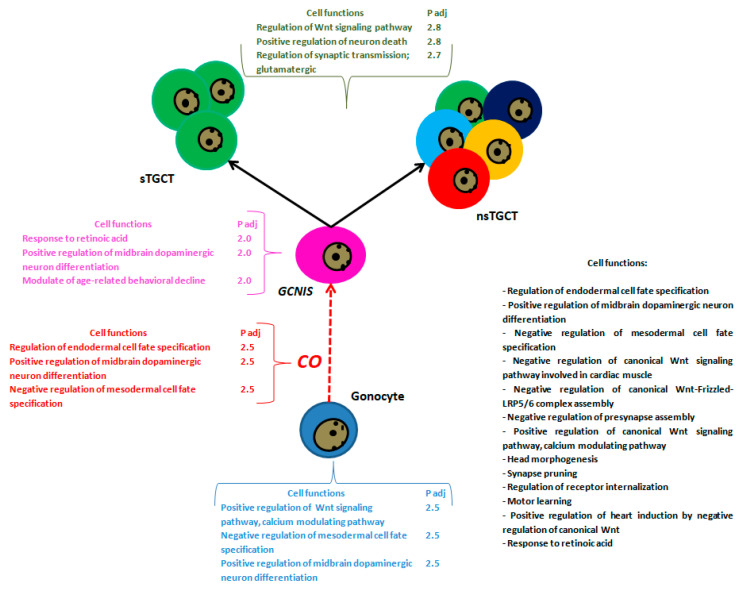
Functional analysis of the genes regulated by the miRNAs described in gonocytes, CO, *GCNIS*, and TGCT. The image shows a summary of the three main biological processes regulated by these miRNAs in gonocytes (blue), CO (red), *GCNIS* (pink), and TGCT (green), generated by GeneCodis 4 tool [111]. Finally, in black is shown the biological processes in common between the four stages regulated by these miRNAs.

Interestingly, during the passage from the gonocyte towards *GCNIS* and the development of the TGCT, we can consistently find alterations in the expression of several miRNAs, particularly in the expression of the hsa-miR-371-373 cluster and hsa-miR-367, which are consistently associated with germ cells. However, whether the mentioned alterations are maintained in other risk conditions, such as CO, as shown in Figure 3, is unknown.

## 4. miRNAs as Potential Serum Markers of TGCT

For cancer diagnosis, biopsy is still the gold standard, which is an invasive and expensive procedure. Therefore, there is a need to look for alternative techniques, such as the use of biological fluids, which represent a non-invasive detection method for sample collection with low cost [112,113]. In this sense, miRNAs have been reported as new diagnostic and prognostic biomarkers in both tissues and body fluids [114]. They are highly stable in the extracellular fluid and can withstand unfavorable physiological conditions such as freezing, thawing, long-term storage, changes in pH, and extreme temperature variations [115]. This is because some miRNAs are encapsulated in exosomes, apoptotic bodies, lipid vesicles, high-density lipoproteins (HDL), and nucleophosmin 1 (NPM1), while others bind to AGO 1 and 2 proteins [24]. The conventional biomarkers used for the diagnosis of TGCT are AFP, which is found at high levels mainly in yolk sac tumors, and hCG, which is elevated in choriocarcinoma. These biomarkers present low sensitivity and specificity, since high levels can be induced by chemotherapy, use of marijuana in the case of hCG, and liver disease in AFP, which can change the expression levels depending on the progression of the disease. Therefore, this limits their use as reliable serum biomarkers, in addition to the fact that only 60% of patients with TGCT show an increase in these markers [115]. In a study conducted by Gillis et al. [116], they demonstrated that hsa-miR-371-373 and hsa-miR-367, when combined as biomarkers, presented sensitivity of 98%, while AFP and hCG presented lower sensitivity, between 36% and 57%, respectively. This demonstrates that miRNAs are superior biomarkers for the diagnosis of TGCT compared to conventional markers. These miRNAs are found at high levels in both the testicular tissue and blood serum of TGCT patients compared to healthy men. In addition, it has been shown that, after orchiectomy in patients with TGCT, the expression levels of these miRNAs decrease [115]. Furthermore, it has been reported that the expression variability of these miRNAs can be used as a biomarker, due to the high sensitivity and ease of detection; the hsa-miR-371-373 group and hsa-miR-367 exhibit potential to be biomarkers for diagnosis, prognosis, and cancer therapy response evaluation [18,108].

## 5. Application of Therapies Using miRNAs

Since miRNAs in blood and other body fluids can be easily detected using non-invasive techniques, they are considered a new generation of biomarkers for various pathologies, including cancer [112,113].

It was recently shown that the regulation of specific miRNA alterations using miRNA mimics (mimic) or miRNA antagonists (antagomiR) can normalize the gene regulatory network and signaling pathways and reverse phenotypes in cancer cells [117]. This is based on the fact that miRNAs can regulate hundreds of genes, which is why they have been used in several studies as targets for therapy against various pathologies [118]. Several mimic and antimiR have been investigated. For miRNA delivery in the clinic, various delivery systems have been tested, such as viral and non-viral vectors; inorganic nanoparticles such as carbon, gold, and silica; lipid nanoparticles, and polymeric complexes [119].

AntimiRs can reduce the function of aberrantly expressed miRNAs, while mimics can increase the expression levels of a specific miRNA, resulting in the suppression of gene expression [120]. AntimiRs are modified antisense oligonucleotides with a sequence complementary to their target miRNA, so they can interfere in miRNA processing, prevent it from binding to RISC, or bind to mature miRNAs in RISC [121]. The antimiR must have a high affinity for its target gene, high specificity, resistance to nucleases, and low toxicity [122]. Locked nucleic acid (LNA)-modified antimiR (LNA–antimiR) are chemically modified oligonucleotides used to detect miRNAs and to bind to the miRNA seed sequence to inhibit its functions, producing increased expression of its target mRNAs [122]. LNAs can be administered by intraperitoneal injections; to date, no acute or chronic toxic effects have been observed in laboratory animals [123]. Another type of antimiR is created by conjugating cholesterol to the 3′ end and O-Me oligonucleotides with a phosphorothioate linkage at the 5′ terminal region to prevent their degradation and increase their binding affinity to silence miRNAs [123]. Additionally, there are the so-called miRNA sponges, which are short oligonucleotides that carry a miRNA binding site and inhibit the aberrant expression of miRNAs by binding to them in a total or partial complementary manner; these can block a whole family of miRNAs with similar targets [124].

In contrast, mimics are composed of a strand identical to the mature miRNA that can be loaded onto the RISC complex and can act as an endogenous miRNA, resulting in the inhibition of gene expression [122]. In addition to antimiRs and mimics, there are other techniques used, such as the masking of miRNAs (miR mask). These are composed of single-chain 2′-O-methyl modified antisense oligonucleotides complementary to the binding site for an endogenous miRNA that binds to the target mRNA with higher affinity, thereby blocking the miRNA’s access to its binding site [119]. In short, miRNA mimics and antagonists, since they are oligonucleotides of small molecular weight, are easier to administer to target cells. Until now, studies have only been carried out in laboratory animals, being effective in rodents. However, strategies to improve tissue-specific delivery, stability, and cellular uptake are still required for future therapy success [120].

## 6. Conclusions

In recent years, the participation and function of various miRNAs in testicular germ cells have been investigated, among which the hsa-miR-371-373 and hsa-miR-302/367 clusters stand out. Various studies have found overexpression of the aforementioned clusters in the gonocyte, the *GCNIS*, and TGCT. To date, it has been described that some patients with CO present persistence of gonocytes and their characteristic protein expression after 6 months of age, which supports the theory of the gonocyte as a target for malignancy. Therefore, it could be inferred that the hsa-miR-371-373 and hsa-miR-302/367 clusters could actively participate in the transformation of the gonocyte towards *GCNIS* and the development of TGCT in adulthood. These miRNAs have not yet been described in pediatric patients with CO, but knowledge of their expression levels could provide insights into the etiology of this malformation and how some patients develop testicular neoplasia.

The results of the hsa-miR-371-373 and hsa-miR-302/367 clusters are promising; thus, in the future, they could be used as specific diagnostic and prognostic biomarkers of TGCT and especially in patients at risk, such as pediatric patients with CO.

## Figures and Tables

**Table 1:** Differential expression of miRNAs in CO.

**Downregulated**		
**miRNA**	**log2FoldChange**	***p* adj**
hsa-miR-3663-5p	−4.426	2.39 × 10^−10^
hsa-miR-1233-3p	−4.228	1.84 × 10^−8^
hsa-miR-552-5p	−4.056	1.21 × 10^−10^
hsa-miR-449b-5p	−3.973	5.26 × 10^−13^
hsa-miR-7153-5p	−3.813	5.18 × 10^−8^
hsa-miR-122-5p	−3.791	1.60 × 10^−9^
hsa-miR-552-3p	−3.761	2.31 × 10^−9^
hsa-miR-449a	−3.741	5.97 × 10^−11^
hsa-miR-122-3p	−3.722	0.0017
hsa-miR-34b-5p	−3.688	3.56 × 10^−9^
hsa-miR-449c-5p	−3.638	1.93 × 10^−12^
hsa-miR-34c-5p	−3.554	5.26 × 10^−13^
hsa-miR-449c-3p	−3.441	0.0011
hsa-miR-375	−3.409	9.99 × 10^−18^
hsa-miR-3663-3p	−3.385	9.63 × 10^−6^
hsa-miR-7159-5p	−3.259	5.29 × 10^−5^
hsa-miR-449b-3p	−3.212	2.75 × 10^−6^
hsa-miR-4700-5p	−3.209	0.0043
hsa-miR-522-3p	−3.153	1.46 × 10^−9^
hsa-miR-1273a	−3.118	2.44 × 10^−8^
hsa-miR-1295a	−3.076	0.0005
hsa-miR-34b-3p	−2.971	2.16 × 10^−7^
hsa-miR-1283	−2.798	2.41 × 10^−7^
hsa-miR-3150b-3p	−2.768	0.0203
hsa-miR-4423-3p	−2.703	0.0023
hsa-miR-6507-5p	−2.699	0.0049
hsa-miR-7154-5p	−2.647	0.0253
hsa-miR-517c-3p	−2.639	9.92 × 10^−10^
hsa-miR-3925-3p	−2.613	0.0025
hsa-miR-515-5p	−2.600	8.84 × 10^−10^
**Upregulated**		
hsa-miR-7151-3p	2.634	0.0137
hsa-miR-376a-2-5p	2.202	0.0109
hsa-miR-1224-5p	2.193	0.0024
hsa-miR-1299	1.958	5.73 × 10^−5^
hsa-miR-142-5p	1.898	0.0060
hsa-miR-543	1.869	0.0004
hsa-miR-487a-3p	1.865	0.0079
hsa-miR-584-3p	1.830	0.0060
hsa-miR-665	1.799	0.0362
hsa-miR-134-3p	1.778	0.0130
hsa-miR-369-3p	1.692	0.0008
hsa-miR-377-3p	1.665	0.0113
hsa-miR-33a-5p	1.665	0.0113
hsa-miR-376a-3p	1.602	0.0016
hsa-miR-758-3p	1.589	0.0020
hsa-miR-654-3p	1.588	0.0004
hsa-miR-134-5p	1.558	0.0017
hsa-miR-889-3p	1.552	0.0052
hsa-miR-127-3p	1.549	0.0007
hsa-miR-1185-1-3p	1.539	0.0110
hsa-miR-1185-2-3p	1.534	0.0305
hsa-miR-154-5p	1.516	0.0001
hsa-miR-381-3p	1.511	0.0007
hsa-miR-127-5p	1.511	0.0013
hsa-miR-337-5p	1.510	0.0036
hsa-miR-379-3p	1.508	0.0013
hsa-miR-136-3p	1.506	0.0010
hsa-miR-376c-3p	1.492	0.0015
hsa-miR-495-3p	1.443	0.0016
hsa-miR-376b-5p	1.442	0.0449

**Table 2:** miRNAs that participate in the development of TGCT.

miRNA	Target Genes	Function	References
*cluster*-miR-302	*Akt* *SPRY4* *NR2F2* *CDK2/4* *CyclinD1*	Participates in the maintenance of embryonic stem cell pluripotency and is highly expressed in seminoma. Regulates Akt, which inhibits the expression of other cell cycle inhibitors such as CDK2 and CDK4, and thus accelerates the transition from G1 to S phase. Inhibition of Spry4 in TGCT decreases cell growth and invasion.	[50,94]
hsa-miR-21		They act as oncomiRs and are found at high levels in seminoma and spermatocytic seminoma.	[95]
hsa-miR-221 hsa-miR-222		Elevated in seminoma.	[95]
hsa-miR-146		Downregulated in seminoma and embryonal carcinoma.	[17]
hsa-Let-7	*Lin28*	Downregulated in TGCT.	[96]
hsa-miR-371a-3p		Upregulated in TGCT.	[97]
hsa-miR-372-3p hsa-miR-373-3p	*LATS2*	They act as oncomiRs, inhibiting p53-mediated cyclin-dependent kinase (CDK) by regulating LATS2, a tumor suppressor, allowing tumor growth in the presence of WT p53. They participate in tumorigenesis.	[98]
hsa-miR-142-3p	*PTPN23*	Participates in the pathogenesis of TGCT.	[99]
hsa-miR-125b	*CSF1* *CX3CL1*	Participates as a tumor suppressor in various types of tumors and has functions in proliferation and apoptosis. Found at low levels in TGCT.	[100]
hsa-miR-223-3p	*FBXW7*	It acts as oncomiR, promoting cell growth and inhibiting apoptosis.	[101]
hsa-miR-199a-5p	*PODXL*	Participates in the development of TGCT.	[102]
hsa-miR-383	*IRF1*	High expression in embryonal carcinoma. Regulates cell proliferation by reducing the levels of cyclin D1, CDK2, and p21.	[103]
hsa-miR-26a hsa-Let-7a	*HMGA1*	They inhibit proliferation and motility in seminoma.	[104]
hsa-miR-449a		Found at low levels in TGCT.	[105]
hsa-miR-514a-3p	*PEG3*	They have low expression in seminoma and embryonal carcinoma. They slow down the apoptotic mechanisms of TGCT.	[106]
hsa-miR-199a-3p	*SP1* *DNMT3A*	Participates in aerobic glycolysis, DNA methylation, and low expression in TGCT.	[107]
has-miR-367-3p,		Highly expressed in TGCT.	[108]
hsa-miR-512-3p, hsa-miR-515-518 hsa-miR-525		Highly expressed in embryonal carcinoma	[109]
hsa-miR-301		Expressed in spermatocytic seminoma, yolk sac tumors, and teratoma.	[17]
hsa-miR-17-5p		Expressed in embryonal carcinoma.	[17]
hsa-miR-375-5p		Expressed in teratoma.	[110]

## Data Availability

Data is contained within the article.
